# Supplementary material for: Content-rich biological network constructed by mining PubMed abstracts
Source: BMC Bioinformatics. 2004 Oct 8;5:147. doi: 10.1186/1471-2105-5-147 (PMC528731; doi:10.1186/1471-2105-5-147)
Supplement: Additional File 2 — The original results of the above study (non-essential files are deleted to keep the file size under the limit set by BMC bioinformatics). [file 1471-2105-5-147-S2.bz2 › chilibotAdditionalFile2/dip30/446ID9468473E10452/html/CCR5_CCL8.html]

 


 **CCR5** and **CCL8** 
  
Found 26 abstracts in PubMed, retrieved 26.  
 

 What does Google say? 
 PDF only 
| .edu only 

---

**Interactive relationship** (e.g. stimulation, inhibition, etc)

**Inhibitory relationship**- These results indicated that MCP 2  [ **CCL8** ]  uses  **CCR5**  as one of its functional receptors and is an additional potent natural inhibitor of HIV 1.  Ref: 9468473 J Biol Chem, 1998
- Monocyte chemotactic protein 2  [ **CCL8** ]  activates  **CCR5**  and blocks CD4  **CCR5**  mediated HIV 1 entry replication.  Ref: 9468473 J Biol Chem, 1998
- These data confirm that MCP 2  [ **CCL8** ]  is a ligand for  **CCR5**  on CD4 lymphocytes and can specifically block R5 HIV 1.  Ref: 11930329 J Infect Dis, 2002
- Furthermore, MCP 2  [ **CCL8** ]  inhibited the entry replication of HIV 1ADA in  **CCR5**  293 cells coexpressing CD4.  Ref: 9468473 J Biol Chem, 1998
- As predicted, MCP 2  [ **CCL8** ]  competitively inhibited MIP 1beta binding to HEK293 cells stably transfected with  **CCR5**   **CCR5**  293 cells.  Ref: 9468473 J Biol Chem, 1998
- Monocyte chemoattractant protein 2  [ **CCL8** ]  CC chemokine ligand 8  [ **CCL8** ]  inhibits replication of human immunodeficiency virus type 1 via CC chemokine receptor 5  [ **CCR5** ] .  Ref: 11930329 J Infect Dis, 2002
- Furthermore, MCP 2  [ **CCL8** ]  inhibited the binding of the M tropic HIV 1 gp120 envelope glycoprotein to  **CCR5**  and HIV 1 infection of peripheral blood mononuclear cells.  Ref: 9790730 Cell Immunol, 1998
- MCP 2  [ **CCL8** ]  is a potent inhibitor of HIV 1 by virtue of its high affinity binding to the receptor  **CCR5** , one of the major coreceptors for HIV 1.  Ref: 11087354 Biochemistry, 2000
**Neutral relationship**- Furthermore, MCP 2  [ **CCL8** ]  induces a robust, pertussis toxin sensitive calcium flux in primary lymphocytes, and cross desensitization studies indicate that MCP 2  [ **CCL8** ]  acts via  **CCR5** .  Ref: 11930329 J Infect Dis, 2002
- We found that MCP 2  [ **CCL8** ]  could bind to  **CCR5**  transfectants with high affinity and cross compete effectively with RANTES, MIP 1alpha, and MIP 1beta.  Ref: 9790730 Cell Immunol, 1998
- Confocal microscopy indicates that MCP 2  [ **CCL8** ]  caused remarkable and dose dependent internalization of  **CCR5**  in  **CCR5**  293 cells.  Ref: 9468473 J Biol Chem, 1998
- MCP 2  [ **CCL8** ]  also bound to and induced chemotaxis of  **CCR5**  293 cells with a potency comparable with that of MIP 1beta.  Ref: 9468473 J Biol Chem, 1998
- In an effort to further characterize the binding properties of MCP 2  [ **CCL8** ]  on leukocytes, we observed that MCP 2  [ **CCL8** ] , but not MCP 1, effectively competed with MIP 1beta for binding to monocytes, suggesting that MCP 2  [ **CCL8** ]  may interact with  **CCR5** .  Ref: 9468473 J Biol Chem, 1998
- MCP 2  [ **CCL8** ]  is a true agonist for  **CCR5** , eliciting a robust chemotactic response in  **CCR5**  transfectants similar to that of the three known  **CCR5**  ligands and exhibiting cross desensitization with RANTES in the Ca2 flux response.  Ref: 9790730 Cell Immunol, 1998
- **CCR5**  has an expanded ligand binding repertoire and is the primary receptor used by MCP 2  [ **CCL8** ]  on activated T cells.  Ref: 9790730 Cell Immunol, 1998
- accepted 8 5 98.  **CCR5**  Has an Expanded Ligand Binding Repertoire and Is the Primary Receptor Used by MCP 2  [ **CCL8** ]  on Activated T Cells.  Ref: 9758698 Cell Immunol< MedlineTA>Cell Immunol, 1998
- **CCR5**  is a G protein coupled receptor for RANTES, MIP 1a, MIP 1b and MCP 2  [ **CCL8** ]  that functions as the front line coreceptor for human immunodeficiency virus type 1 infection.  Ref: 12837756 J Biol Chem< MedlineTA>J Biol Chem, 1998
- **CCR5**  is most closely related to CCR2b, another chemokine receptor that responds to monocyte chemoattractant protein MCP 1, MCP 2  [ **CCL8** ] , MCP 3, and MCP 4.  Ref: 9312096 J Biol Chem, 1997
- **CCR5**  is a functional receptor for MIP 1alpha, MIP 1beta, RANTES regulated on activation normal T cell expressed, MCP 2  [ **CCL8** ] , and MCP 4 and constitutes the main coreceptor for macrophage tropic human and simian immunodeficiency viruses.  Ref: 10574939 J Biol Chem, 1999
- **CCR5**  is a G protein coupled receptor activated by the chemokines RANTES regulated on activation normal T cell expressed and secreted, macrophage inflammatory protein 1alpha and 1beta, and monocyte chemotactic protein 2  [ **CCL8** ]  and is the main co receptor for the macrophage tropic human immunodeficiency virus strains.  Ref: 11278962 J Biol Chem, 2001
- **CCR5**  is a G protein coupled receptor responding to four natural agonists, the chemokines RANTES regulated on activation normal T cell expressed and secreted, macrophage inflammatory protein MIP 1 alpha, MIP 1 beta, and monocyte chemotactic protein MCP 2  [ **CCL8** ] , and is the main co receptor for the macrophage tropic human immunodeficiency virus strains.  Ref: 12411445 J Biol Chem, 2003
- MIP 1alpha, MIP 1beta, RANTES, MCP 2  [ **CCL8** ]  and MCP 4 were able to stimulate increases in intracellular calcium ions via activation of  **CCR5**  whereas MCP 3 was without effect.  Ref: 11861332 Br J Pharmacol, 2002
- The roles of  **CCR5**  in excitotoxic injury in  **CCR5**  deficient mice are compensated by increased CCR2 and CCR3 expression, which share the common MCP 2  [ **CCL8** ]  ligand with  **CCR5** .  Ref: 12807425 J Neurochem, 2003

**Non-interactive relationship** (e.g. studied together, co-existance, homology, etc.)

- Recently we reported that the chemokines MIP 1alpha, MIP 1beta, and RANTES secreted by BM derived CD34 cells may compete with the macrophagotropic HIV R5 HIV strain for the  **CCR5**  coreceptor.In this study we extended our previous observations and examined various lympho hematopoietic CD34 cells isolated from thymus Th, cord blood CB, mobilized peripheral blood mPB, and BM for the expression of beta chemokines binding to  **CCR5** , i.e., MIP 1alpha, MIP 1beta, RANTES, MCP 2  [ **CCL8** ] , MCP 3, and MCP 4, and the alpha chemokine SDF 1 binding to CXCR4 as these chemokines may compete with the R5 and X4 HIV strains, respectively, for entry into cells.We found that Th, CB, mPB, and BM derived CD34 cells express mRNA transcripts for all the beta chemokines tested but not for SDF 1.  Ref: 11146155 Exp Hematol, 2000
- Thus, although MCP 2  [ **CCL8** ]  can bind to and signal through CCR1, CCR2b, and  **CCR5** , among which both CCR2 and  **CCR5**  are expressed at high levels on activated T cells, it appears to preferably utilize  **CCR5**  on these cells.  Ref: 9790730 Cell Immunol, 1998
- **CCR5** , a receptor for chemokines MIP 1alpha LD78alpha, MIP 1beta, RANTES, and MCP2  [ **CCL8** ] , is of particular importance in vivo as polymorphisms in this gene affect HIV infection and rate of progression to AIDS.  Ref: 10364178 J Biol Chem, 1999
- We investigated the effects of a number of naturally occurring chemokines MIP 1alpha, MIP 1beta, RANTES, MCP 2  [ **CCL8** ] , MCP 3, MCP 4 on different processes linked to the chemokine receptor  **CCR5**  in recombinant CHO cells expressing the receptor at different levels.  Ref: 11861332 Br J Pharmacol, 2002
- A number of inflammatory CC chemokines, including MIP 1 alpha, MIP 1 beta, RANTES, MCP 2  [ **CCL8** ] , and HCC 1 9 74 act as  **CCR5**  agonists, while MCP 3 is a natural antagonist of the receptor.  Ref: 12402506 Receptors Channels, 2002
- The chemokines Mig CXCR3 ligand, TARC CCR4 ligand, and MCP 2  [ **CCL8** ]   **CCR5**  ligand were detected in intratumoral blood vessels and histiocytes.  Ref: 10887135 Blood, 2000
- More importantly, we found that MCP 2  [ **CCL8** ]  could bind and elicit chemotaxis in CD3 activated and IL 2 maintained T cells, and most of these functions could be specifically inhibited by the anti  **CCR5**  mAb 2D7.  Ref: 9790730 Cell Immunol, 1998
- In order to evaluate if these cells are infectabile by human immunodeficiency virus HIV and could be employed as a model in HIV research we exposed these cell lines to X4 T cell tropic and R5 macrophage tropic and subsequently tried to correlate their infectability with i level of chemokine coreceptor CXCR4 and  **CCR5**  expression, ii coreceptor functionality calcium flux, chemotaxis and phosphorylation of MAPK p42 44 and AKT and iii endogenous expression and secretion of HIV related chemokines which compete with the virus for binding to CXCR4 SDF 1 CXCL12 or  **CCR5**  MIP 1beta CCL4, MIP 1alpha CCL3, RANTES CCL5, MCP 2  [ **CCL8** ]   **CCL8** , MCP 3 CCL7 and MCP 4 CCL13.  Ref: 11737246 Eur J Haematol, 2001
